# The Institutionalisation of Digital Public Health: Lessons Learned from the COVID-19 App

**DOI:** 10.1017/err.2020.47

**Published:** 2020-04-28

**Authors:** Ciro CATTUTO, Alessandro SPINA

**Affiliations:** * Associate Professor in the Computer Science Department Computer of the University of Turin (Italy) and Principal Scientist and Research Area Coordinator at the ISI Foundation of Turin.; ** Member of the Legal Service, European Commission (Brussels) and Visiting Lecturer at the Faculty of Law of the University of Fribourg (Switzerland); email: alessandro.spina@protonmail.com.

## Abstract

Amid the outbreak of the SARS-CoV-2 pandemic, there has been a call to use innovative digital tools for the purpose of protecting public health. There are a number of proposals to embed digital solutions into the regulatory strategies adopted by public authorities to control the spread of the coronavirus more effectively. They range from algorithms to detect population movements by using telecommunications data to the use of artificial intelligence and high-performance computing power to detect patterns in the spread of the virus. However, the use of a mobile phone application for contact tracing is certainly the most popular.

## I. Introduction

Amid the outbreak of the SARS-CoV-2 pandemic, there has been a call to use innovative digital tools for the purpose of protecting public health. There are a number of proposals to embed digital solutions into the regulatory strategies adopted by public authorities to control the spread of the coronavirus more effectively. They range from algorithms to detect population movements by using telecommunications data to the use of artificial intelligence and high-performance computing power to detect patterns in the spread of the virus.^1^ However, the use of a mobile phone application for contact tracing^2^ is certainly the most popular.

These proposals, which have a very powerful persuasive force and have apparently contributed to the success of public health responses in a few Asian countries, also raise questions and criticisms, particularly with regards to the risks that these novel digital surveillance systems pose for privacy and in the long term for our democracies.

With this short paper, we would like to describe the pattern that has led to the institutionalisation of digital tools for public health purposes. By tracing their origins to “digital epidemiology”, an approach that originated in the early 2010s, we will expose that, whilst there exists limited experimental knowledge on the use of digital tools for tracking disease, this is the first time in which they are being introduced by policy-makers into the set of non-clinical emergency strategies to a major public health crisis. As reflected in the public debate about the design and implementation of the contact tracing app for COVID-19, the novelty of using digital tools in public health brings in a set of questions concerning not only their alleged effectiveness, but also their compatibility with privacy right and fairness. The institutionalisation of digital tools for COVID-19 is therefore taking place within a system of public governance that is unprepared to tackle the ethical, social and legal challenges of these technologies.

The aim of this paper is to demonstrate, with reference to the controversies of the COVID-19 contact tracing app, that the institutionalisation of digital tools for public health requires novel institutional mechanisms to manage their complexity and risks. Our conclusion is that, like in the regulatory models of other risk technologies such as medicines or genetically modified organisms (GMOs), we need to have transparency and public oversight of digital public health.

## II. The origins of digital epidemiology

From a scientific point of view, the current efforts to leverage the public health measures on digital strategies should be seen in the broader context of digital epidemiology.^3^ What is digital epidemiology? The goal of epidemiology is to understand the patterns of disease and health dynamics in populations, as well as the causes of these patterns, and to use this understanding to mitigate and prevent disease and to promote health. The goal of digital epidemiology is exactly the same, with the main difference being that whilst epidemiology uses traditional data collected by health protection agencies and healthcare providers (eg patient medical records, death certificates, disease registries), digital epidemiology uses digital data, and often data that were not originally collected for health applications^4^ (eg search engine queries mentioning medical symptoms for population-scale disease surveillance).

Perhaps one of the first and most well-known examples of digital epidemiology is Google Flu Trends (GFT), which used symptomatic search queries for the purpose of syndromic tracking of influenza-like illnesses.^5^ Although the technical merit of the specific tool was subsequently criticized,^6^ it did have a lasting impact in creating awareness about new opportunities for the secondary use of digital data, and it paved the way to a rich field of research. Another example of digital epidemiology approaches is the use of telecommunications data to examine the movements of people and their influence on infectious disease dynamics. For example, mobile phone call records were analysed as indicators of travel patterns in an African country, and this provided understanding of malaria spread caused by human mobility.^7^ In another study, the position data of subscriber identity module (SIM) cards from the largest mobile phone company in Haiti were used to estimate the magnitude and trends of population movements following the 2010 Haiti earthquake and cholera outbreak.^8^ All of the above examples show that the findings of digital epidemiology are based on data generated outside of the public health system (ie data that were not generated with the primary purpose of doing epidemiology).^9^ Such a secondary use of data, often privately held at the origin, underpins both opportunities and specific challenges related to digital epidemiology. The other defining character of digital epidemiology is its use of computational and analytical methodologies originating in computer science, such as machine learning, computer vision, natural language processing and more.^10^

Other success stories of digital epidemiology have been based on a more participatory/empowerment model of disease surveillance. In the case of Influenzanet,^11^ FluNearYou and Flutracking, for example, citizens acted as self-reporting volunteers and shared self-reported information on symptoms to allow for better comprehension of the determinants of influenza-like illness.^12^

With regards to the measures adopted or being contemplated against COVID-19, we are seeing proposals that rely on data generated outside of public health systems, such as the use of telecommunications for tracking population movements for the classical epidemiological goals of understanding the causes and spread of a virus. However, and this is perhaps the breakthrough novelty of the current circumstances, there is also an institutionalisation of the use of software applications (eg a mobile phone app) that are designed to generate data that not only would be relevant from an epidemiological point of view, but also would be used by public authorities effectively as a form of public health risk management.

Whilst the examples of digital epidemiology carried out in the past decade such as GFT, Influenzanet or HealthMap demonstrated the potential of using digital data as an increasingly valuable proxy for human behaviour for the purpose of scientific research, the proposed integration of these techniques within public health systems of interventions that are “born digital” raises a completely different set of governance and regulatory challenges.

In fact, although digital epidemiology as a scientific domain is a decade old, its integration in public health systems has been, before the outbreak of the SARS-CoV-2 pandemic, almost non-existent. It is true that, for example, looking at the European level, there had been instances in which public health authorities explored these new methods of digital intelligence, particularly with regards to the monitoring of digital sources in order to detect potential disease outbreaks in a timely manner^13^ or with regards to the use of mobile apps or social media for pharmacovigilance purposes.^14^ But in the context of the COVID-19 pandemic crisis, the focus has markedly shifted towards a fast uptake of digital tools, and in particular the mobile phone app for contact tracing, in public health strategies.

## III. The institutionalisation of the COVID-19 contact tracing app

Similarly to the many research projects exploring the application of digital technologies to disease detection, a number of projects used mobile phone signals to measure, understand and predict how individuals change their social behaviour in response to infectious disease.^15^

Software applications such as apps can be used to make mobile phones perform certain recording tasks that are potentially useful for aiding contact tracing, particularly given the fact that, in the case of COVID-19, the disease incubation period is relatively long and individuals that are pre-symptomatic or asymptomatic can spread the virus. Additionally, an app offers a scalable and easily deployable complement to the traditional system of retrieval of information and notification of potentially infected individuals. Therefore, a mobile phone app can be used as a tool for performing critical epidemiological functions,^16^ and it has been part of the public health measures adopted during the pandemic outbreak by a number of Asian countries for tracing individuals at risk of developing the disease.

Therefore, by looking at the comparative efforts at the adoption of a contact tracing app and the problems caused by the lack of a pre-existing common regulatory framework, it is possible to gain insights into the challenges of digital public health.

Firstly, it should be observed that while the idea of a digital solution for contact tracing has officially become part of the envisioned European response to COVID-19,^17^ there has been no attempt to create a European Union (EU)-wide digital tool, and each Member State is taking its own approach. Recognising the importance of this instrument but also the possibility for multiple national implementations, the European Commission has published a set of guidance and recommendations on the technical and legal aspects for the development of a contact tracing app^18^ that the Member States should respect.

Secondly, the processes used by each Member State for the choice of the app model also reveal striking differences in terms of their transparency and the role played by technical expertise and that of the public or by the national parliaments. In France, for example, President Emmanuel Macron referred to the use of a digital application in the strategy of lifting the lockdown measures as one of the measures regarding which he invited the French Parliament to debate and approve.^19^ Yet, it is not clear what type of contribution will finally be given by the parliamentary assembly.^20^ In Italy and in The Netherlands, their governments have used a procurement-type procedure by launching a public call for app developers^21^ to submit technical proposals for a model of a contact tracing app, and these governments have taken their decisions on the basis of an assessment of the models performed by experts appointed for this task. In the UK, the contact tracing app is being developed by the digital transformation service of the National Health Service (NHSX).^22^

Thirdly, it is envisaged that, before being rolled out to the public, the contact tracing app is to be tested or used under controlled conditions.^23^ Testing of the app is considered useful for assessing the effectiveness of the proposed intervention, but also for fine-tuning critical parameters for alerting potential exposed individuals and for avoiding high rates of false positives that could overwhelm the logistical capabilities of the public health service downstream. However, there are no commonly agreed methodologies for assessing the risks or measuring *ex ante* the desired outcomes of digital interventions, and therefore there will be very little evidence available before the app is being promoted as a tool for contact tracing.

Therefore, the SARS-CoV-2 pandemic and the response from public authorities can be seen as a turning point for digital epidemiology and for the future role that digital tools will play in public health. It is too early to say whether this will be, ultimately, a success or failure; however, regardless of the final outcomes of these specific projects, we believe that there are some important lessons to learn from this case. In particular, the public debate and the vexed governance process that has accompanied its emergence can provide important lessons for studies of risk regulation and, more generally, for future policy-making in this area.

In the considerations that follow, we will summarise a number of points concerning the risks stemming from the use of the contact tracing app that are generalisable to other digital tools for public health.

## IV. The framing of risks of digital technologies for public health

It should first be observed that the introduction of a mobile phone app as a solution to implementing an effective form of contact tracing has been marred by incorrect assumptions and false dichotomies, the first and foremost being the one that accepts that there is a trade-off between the need to respect privacy/data protection and public health. For example, in some extreme instances, a plea has been made for a “temporary” suspension of privacy rights in order to allow the use of digital instruments of surveillance to fight the spread of the virus.^24^ On the other hand, an argument has been made that the use of a mobile phone app for public health purposes is in reality a sort of “Trojan horse” that will make everyone accept more and more intrusive and dystopian forms of digital surveillance in our daily lives. It is certainly true that the implementation of such a privacy-invasive intervention could raise the risk of normalising digital surveillance.^25^ In reality, the massive scale, granularity and quantity of personal data necessary for implementing a process of contact tracing does not prevent the implementation of serious governance and technical mechanisms to limit, to the maximum extent^26^ possible, the interference with the right to privacy and the risk of a “slippery slope” towards generalised digital surveillance. Some of the technical approaches that are being considered are designed around privacy-by-design principles that include data minimisation: for example, decentralised digital contact tracing systems (such as the “DP3T” protocol^27^) store contacts’ pseudonyms locally on a user’s smartphone, and only transfer this information to a central server after a confirmed diagnosis, allowing risk scores of all of the other users to be updated in a decentralised computation so that no information about negative cases is ever collected and no centralised view of the (social) proximity graph is constructed.

An incorrect assumption is that digital tools for public health, similarly to apps developed by corporations for commercial purposes, should seek to obtain as much personal data as possible, even though there is no clear justification underpinning the acquisition of such data. In reality, this seems to be a misconceived approach. On the contrary, the collection of data that are not necessary for strict public health purposes increases the risk of “function creep”: for example, whether geo-localisation data collected by a contact tracing app could be used for the purpose of enforcement of public order rules, or when it is proposed to mix the contact tracing purpose of the app with other functionalities, such as symptom reporting or telemedicine, which are not strictly necessary from an epidemiological perspective, but may serve other public health purposes, making it difficult to reason about the proportionality and risk/benefit balances of the primary function.

Another false dichotomy rests on the idea that, in order to be effective, the contact tracing app must be made mandatory (ie it has to be pushed into people’s lives rather than “opted into”). In reality, this simplistic approach triggers risks of all sorts of adversarial scenarios and new “attack surfaces”, leading to the possibility of new cybersecurity threats, organised protests and boycotts, spamming and flooding of the system.^28^ On the contrary, a more collaborative, voluntary and interactive approach would enhance the empowerment and autonomy of citizens^29^ and is more likely to lead to a bigger response by the population in terms of app downloads. It is perhaps relevant here to mention that even non-coercive, “nudging” techniques to promote the use of the app could negatively impact individual liberty, particularly with regards to vulnerable groups or individuals.^30^

Despite the fact that there is no available evidence of the effectiveness of digital contact tracing, some outspoken proponents of the contact tracing app seem to hold a misconceived belief that a complex and multi-layered issue such as the containment of COVID-19 in our societies can mainly be tackled by computer engineering and digital solutions. This type of technological solutionism raises a series of questions linked not only to the possibility of privacy violations, but also to social fairness. By ruling out or downplaying the current digital divide and the digital illiteracy of large parts of the population, the idea of a digital tool as the exclusive way to trace contacts of potentially infected persons during a pandemic would bring about unfair social discrimination resulting in different forms of health protection for those that have and those that do not have access to digital technologies.

Finally, it should also be acknowledged that a form of power asymmetry exists between public authorities and the big technology companies that own the app platforms. The latter are effectively in control of the digital infrastructure on which the digital tools designed for public health are supposed to operate and therefore can make far-reaching policy decisions. Additionally, in the case of the contact tracing app, based on the public statement by Apple and Google,^31^ it is envisaged that the contact tracing capabilities will be migrated into the operating systems of mobile phones (which remain the proprietary assets of private companies). This might have security and technical advantages for the functionality of the app; however, it also reduces the visibility of the technical implementation details. In summary, the public health function of the app can be designed and shaped by governments; however, these apps can only work via privately held platforms that ultimately maintain an important “gatekeeper” function.^32^

## V. Concluding remarks

Since the first experimental applications of digital epidemiology, it could have been easily imagined that the concrete and visible digital transformation of our economies and societies would have led to a call for public authorities to make use of digital tools for public health. The current pandemic outbreak has catalysed the institutionalisation process of digital tools for public health and clearly highlighted current gaps in the public governance system.

On the one hand, the exploitation of digital data for public interest goals such as the fight against COVID-19 is increasingly accepted not only by the scientific community, but also by the public at large.^33^ On the other hand, as we have illustrated with the contact tracing app for COVID-19, the system of public oversight of digital tools for public health is not yet prepared to address fully the ethical, social and legal challenges of these technologies. This creates a haphazard situation of potential conflict between technical expertise and public perception of risks, a situation that is widely known and examined in many domains of risk regulation.

Therefore, the institutionalisation of digital tools for public health also requires an institutionalisation of the regulatory framework for the design and deployment of these tools. The regulatory model of other risk technologies such as GMOs or medicines can offer a valid and proven blueprint, particularly regarding the procedures for the involvement of expert bodies, the mechanisms of participation and involvement of the public, the methodologies for assessing their effectiveness and risks^34^ and finally the transparency requirements and responsibilities of the oversight bodies.^35^

## Footnotes

1 Cf <https://ec.europa.eu/digital-single-market/en/content/digital-technologies-actions-response-coronavirus-pandemic>.

2 “Contact tracing” is a public health intervention based on monitoring contacts after exposure to an infected person and aimed essentially to prevent further transmission of a virus: for more details, see <https://www.who.int/news-room/q-a-detail/contact-tracing>.

3 M Salathé et al, “Digital Epidemiology” (2012) 8(7) PLoS Computational Biology e1002616.

4 A representative model of the structured effort to gather and analyse web-based signals for the purpose of disease surveillance is a system called HealthMap: JS Brownstein, CC Freifeld, BY Reis and KD Mandl, “Surveillance Sans Frontières: Internet-Based Emerging Infectious Disease Intelligence and the HealthMap Project” (2008) 5(7) PLoS Medicine e151.

5 J Ginsberg, MH Mohebbi, RS Patel, L Brammer, MS Smolinski and L Brilliant, “Detecting Influenza Epidemics Using Search Engine Query Data” (2009) 457(7232) Nature 1012.

6 D Lazer et al, “The Parable of Google Flu: Traps in Big Data Analysis” (2014) 343(6176) Science 1203.

7 A Wesolowski et al, “Quantifying the Impact of Human Mobility on Malaria” (2012) 338(6104) Science 267.

8 L Bengtsson et al, “Improved Response to Disasters and Outbreaks by Tracking Population Movements with Mobile Phone Network Data: A Post-Earthquake Geospatial Study in Haiti” (2011) 8(8) PLoS Medicine e1001083.

9 M Salathé, “Digital Epidemiology: What Is It and Where Is It Going*?*” (2018) 14(1) Life Sciences, Society and Policy 1.

10 Various studies have used computer vision algorithms to speed up the process of disease detection: S Robertson et al, “Digital Image Analysis in Breast Pathology – From Image Processing Techniques to Artificial Intelligence” (2018) 14 Translational Research 19; M Usman, “Retrospective Motion Correction in Multishot MRI Using Generative Adversarial Network” (2020) 10(1) Scientific Reports 1; R Singh, “Deep Learning in Chest Radiography: Detection of Findings and Presence of Change” (2018) 13(10) PLoS One e0204155.

11 SP Van Noort, CT Codeço, CE Koppeschaar, M Ranst, D Paolotti and MG Gomes, “Ten-Year Performance of Influenzanet: ILI Time Series, Risks, Vaccine Effects, and Care-Seeking Behaviour” (2015) 13 Epidemics 28.

12 MS Smolinski, AW Crawley, JO Olsen, T Jayaraman and M Libel, “Participatory Disease Surveillance: Engaging Communities Directly in Reporting, Monitoring, Responding to Health Threats (2017) 3(4) JMIR Public Health Surveillance e62.

13 SL Roberts, “Signals, Signs and Syndromes: Tracing [Digital] Transformations in European Health Security” (2019) 10(4) European Journal of Risk Regulation 722.

14 A research project aimed at exploring the potential of new technologies such as mobile apps or social media listening for the purpose of pharmacovigilance by regulatory authorities is the WEB-RADR project; for more information, see <https://web-radr.eu>. For an overview, in general terms, of the ongoing efforts to integrate digital systems into public health strategies, see A Odone et al, “Public Health Digitalization in Europe: EUPHA Vision, Action and Role in Digital Public Health” (2019) 29 European Journal of Public Health 28.

15 For example, the FluPhone project at the University of Cambridge: <https://www.cam.ac.uk/research/news/fluphone-disease-tr.cking-by-app>.

16 L Ferretti et al, “Quantifying SARS-CoV-2 Transmission Suggests Epidemic Control with Digital Contact Tracing”, (2020) Science 10.1126/science.abb6936.

17 Cf the Joint European Roadmap towards lifting COVID-19 containment measures: <https://ec.europa.eu/info/sites/info/files/communication_-_a_european_roadmap_to_lifting_coronavirus_containment_measures_0.pdf>; in particular, by referring to the Recommendation of 8 April 2020 on a common Union toolbox for the use of technology and data to combat and exit from the COVID-19 crisis, particularly concerning mobile applications and the use of anonymised mobility data (C(2020) 2296 final).

18 On 15 April 2020, the European Commission published a document entitled “EU Toolbox for the Use of Mobile Applications for Contact Tracing and Warning”, which originates from a network of Member States’ authorities, supported by the European Commission (the eHealth Network). This document aims at providing practical guidance to Member States for the development of contact tracing apps: the document is available at <https://ec.europa.eu/health/sites/health/files/ehealth/docs/covid-19_apps_en.pdf>. On the same day, the European Commission also published guidance to ensure that contact tracing apps comply with data protection legislation: <https://ec.europa.eu/commission/presscorner/detail/en/ip_20_669>.

19 E Macron, *Adresse aux Français*, 13 April 2020 <https://www.elysee.fr/front/pdf/elysee-module-15482-fr.pdf>, in particular: “*Pour accompagner cette phase, plusieurs innovations font l’objet de travaux avec certains de nospartenaires européens, comme une application numérique dédiée qui, sur la base du volontariat et del’anonymat, permettra de savoir si, oui ou non, l’on s’est trouvé en contact avec une personne contaminée. Vous en avez sûrement entendu parler*”.

20 <https://www.politico.eu/article/macrons-coronavirus-tracking-app-sparks-controversy-in-his-own-camp>.

21 For the Italian “fast call” project led by the Ministry of Digital Innovation, see <https://innovazione.gov.it/innova-per-l-Italia-la-tecnologia-e-l-innovazione-in-campo-contro-l-emergenza-covid-19>; for the Dutch case, see <https://www.rijksoverheid.nl/onderwerpen/coronavirus-app/nieuws/2020/04/11/oproep-om-mee-te-denken-over-apps>. It is also worth mentioning here as an example of experimental governance that the seven app models shortlisted in The Netherlands would then be used for a public “*appathon*”: <https://www.rijksoverheid.nl/onderwerpen/coronavirus-app/nieuws/2020/04/15/ministerie-van-vws-organiseert-digitaal-evenement-voor-beoordeling-corona-apps>.

22 See <https://www.theguardian.com/uk-news/2020/mar/31/nhs-developing-app-to-trace-close-contacts-of-coronavirus-carriers>.

23 See <https://www.bbc.com/news/technology-52381103>.

24 During the pandemic outbreak, the governor of the Italian region of Veneto, Mr Luca Zaia, suggested that privacy law could be temporarily suspended in order to let public health systems trace the contacts of infected people: <https://www.repubblica.it/politica/2020/03/26/news/zaia_sospensione_privacy-252373104>.

25 See, eg, the considerations of V Dubal, “The Expansion of Mass Surveillance to Stop Coronavirus Should Worry Us All”, *The Guardian*, 18 April 2020 <https://www.theguardian.com/commentisfree/2020/apr/18/mass-surveillance-coronavirus-technology-expansion>.

26 See the recommendations of the European Data Protection Board (EDPB) contained in the “Guidelines 04/2020 on the use of location data and contact tracing tools in the context of the COVID-19 outbreak” adopted on 21 April 2020: <https://edpb.europa.eu/our-work-tools/our-documents/guidelines/guidelines-042020-use-location-data-and-contact-tracing_en>.

27 See <https://github.com/DP-3T/documents/blob/master/DP3T%20White%20Paper.pdf>.

28 Security experts R Anderson and B Schneier have already warned about this type of risk; see, respectively, <https://www.lightbluetouchpaper.org/2020/04/12/contact-tracing-in-the-real-world> and <https://www.schneier.com/blog/archives/2020/04/contact_tracing.html>.

29 M Nanni et al, “Give More Data, Awareness and Control to Individual Citizens, and They Will Help COVID-19 Containment” (2020) 13(1) Transactions on Data Privacy 61.

30 Cf the Statement of the IEEE regarding the ethical implementation of artificial intelligence systems (AIS) for addressing the COVID-19 Pandemic: <https://standards.ieee.org/content/dam/ieee-standards/standards/web/documents/other/gieais-covid.pdf>.

31 Cf <https://www.apple.com/newsroom/2020/04/apple-and-google-partner-on-covid-19-contact-tracing-technology>.

32 For example, on 14 March 2020, Apple took the decision to vet any app to be placed in the App Store to ensure that “data sources are reputable and that developers presenting these apps are from recognized entities such as government organizations, health-focused NGOs, companies deeply credentialed in health issues, and medical or educational institutions”: <https://developer.apple.com/news/?id=03142020a>.

33 M Ienca and E Vayena, “On the Responsible Use of Digital Data to Tackle the COVID-19 Pandemic” (2020) 26 Nature Medicine 463.

34 See, Editorial, “Show Evidence That Apps for COVID-19 Contact-Tracing Are Secure and Effective” (2020) 580 Nature 563.

35 The instance of transparency and public reviewability of the source code is included, with other requirements aimed at guaranteeing public oversight of the contact tracing app model, in the Resolution of the European Parliament of 17 April 2020 on EU coordinated action to combat the COVID-19 pandemic and its consequences (2020/2616(RSP)).

